# Folic acid handling by the human gut: implications for food fortification and supplementation^1^,^2^,^3^

**DOI:** 10.3945/ajcn.113.080507

**Published:** 2014-06-18

**Authors:** Imran Patanwala, Maria J King, David A Barrett, John Rose, Ralph Jackson, Mark Hudson, Mark Philo, Jack R Dainty, Anthony JA Wright, Paul M Finglas, David E Jones

**Affiliations:** 1 From the Institute of Cellular Medicine, Newcastle University, Newcastle upon Tyne, United Kingdom (IP, MH, and DEJ); the Institute of Food Research, Norwich Research Park, Norwich, United Kingdom (MJK, MP, JRD, AJAW, and PMF); the Centre for Analytical Bioscience, School of Pharmacy, University of Nottingham, Nottingham, United Kingdom (DAB); and the Department of Radiology, Freeman Hospital, Newcastle upon Tyne, United Kingdom (JR and RJ).

## Abstract

**Background:** Current thinking, which is based mainly on rodent studies, is that physiologic doses of folic acid (pterylmonoglutamic acid), such as dietary vitamin folates, are biotransformed in the intestinal mucosa and transferred to the portal vein as the natural circulating plasma folate, 5-methyltetrahydrofolic acid (5-MTHF) before entering the liver and the wider systemic blood supply.

**Objective:** We tested the assumption that, in humans, folic acid is biotransformed (reduced and methylated) to 5-MTHF in the intestinal mucosa.

**Design:** We conducted a crossover study in which we sampled portal and peripheral veins for labeled folate concentrations after oral ingestion with physiologic doses of stable-isotope–labeled folic acid or the reduced folate 5-formyltetrahydrofolic acid (5-FormylTHF) in 6 subjects with a transjugular intrahepatic porto systemic shunt (TIPSS) in situ. The TIPSS allowed blood samples to be taken from the portal vein.

**Results:** Fifteen minutes after a dose of folic acid, 80 ± 12% of labeled folate in the hepatic portal vein was unmodified folic acid. In contrast, after a dose of labeled 5-FormylTHF, only 4 ± 18% of labeled folate in the portal vein was unmodified 5-FormylTHF, and the rest had been converted to 5-MTHF after 15 min (postdose).

**Conclusions:** The human gut appears to have a very efficient capacity to convert reduced dietary folates to 5-MTHF but limited ability to reduce folic acid. Therefore, large amounts of unmodified folic acid in the portal vein are probably attributable to an extremely limited mucosal cell dihydrofolate reductase (DHFR) capacity that is necessary to produce tetrahydrofolic acid before sequential methylation to 5-MTHF. This process would suggest that humans are reliant on the liver for folic acid reduction even though it has a low and highly variable DHFR activity. Therefore, chronic liver exposure to folic acid in humans may induce saturation, which would possibly explain reports of systemic circulation of unmetabolized folic acid. This trial was registered at clinicaltrials.gov as NCT02135393.

## INTRODUCTION

Naturally occurring dietary folates are a group of water soluble polyglutamate tetrahydrofolate B vitamins (mainly methyltetrahydrofolates and formyltetrahydrofolates) that are vital single carbon donors in human metabolism. A low folate status has been associated with adverse health outcomes. In pregnancy, it is unambiguously associated with increased risk of fetal neural tube defects that can be reduced by periconceptual folic acid supplementation (1). A low folate status has also been associated with elevated plasma homocysteine, which has been a suggested risk factor for cardiovascular disease, stroke, and dementia (2–4), and altered DNA methylation and uracil-induced genomic instability, which may increase risk of colorectal cancer in theory (5) but perhaps not in practice (6). Therefore, an optimal dietary intake of folate is important. An alternative approach, which would give universal benefit, is to fortify food with folic acid. A number of countries, including the United States, Canada, and Chile already have mandatory programs of folic acid fortification of flour (7).

Concerns have been mounting about the safety of a persistent exposure to folic acid that results in the circulation of unmetabolized folic acid (8), including the potential for masking vitamin B-12 deficiency (9) and the acceleration of cognitive decline in the elderly with a low vitamin B-12 status (10, 11). An increase in the incidence of prostate and other cancers was seen in studies performed to address the hypothesis that folic acid supplementation reduces cancer risk, and an increase in overall mortality was seen in patients who were taking folic acid supplements (12–15).

That dietary folate is beneficial but supplemental folic acid may have some detrimental effects is a paradox because both dietary folates and folic acid are taken up by mucosal cells with a similar affinity by the proton-coupled folate transporter (16), and the absorptive mucosa simply rearranges 5-formyltetrahydrofolic acid (5-FormylTHF)^4^ to 5-methyltetrahydrofolic acid (5-MTHF) before transport to the serosal side (17) and transports 5-MTHF unchanged. The generally accepted wisdom (derived from rodent studies) is that physiologic doses of folic acid are biotransformed in the intestinal absorptive mucosa and transferred to the hepatic portal vein as 5-MTHF in the same way as dietary folates (18–20). That this process may also be applicable to humans may have been a misreading of an article that concluded “under physiological conditions only 5-MTHF reaches the blood.” However, the article referred to a study where only a small percentage of ingested folate was folic acid (21). This apparent consensus was challenged by studies that showed a significantly different systemic plasma (labeled) 5-MTHF appearance after the ingestion of single, physiologic doses of stable-isotope–labeled vitamin folates and folic acid (22).

The aim of the current study was to identify the site of biotransformation of folic acid in humans by sampling portal venous blood from subjects with a transjugular intrahepatic porto systemic shunt (TIPSS) in situ who were exposed to orally ingested labeled folic acid or a physiologic dietary folate (formyltetrahydrofolic acid).

## SUBJECTS AND METHODS

### Study design

**FIGURE 1.**
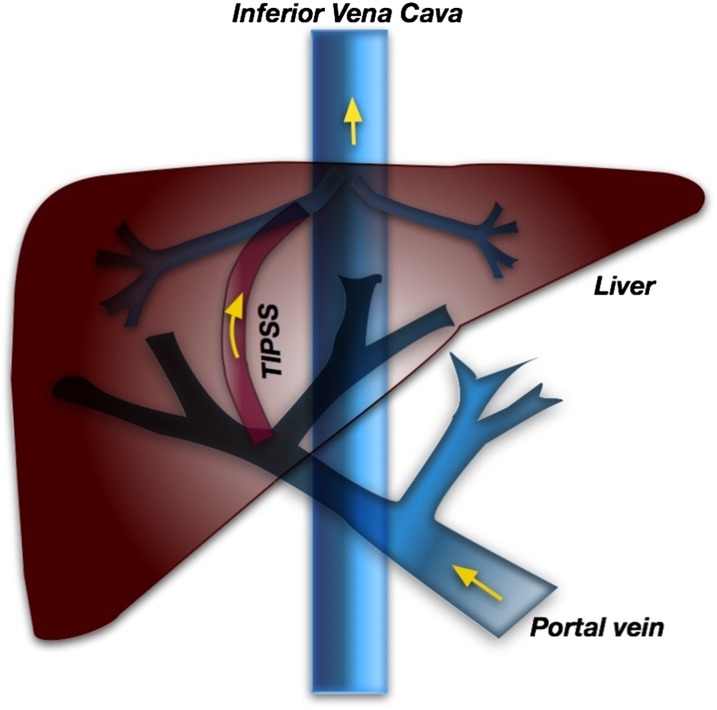
Location of the TIPSS in the liver. TIPSS, transjugular intrahepatic porto systemic shunt.

In the current study, we used an opportunity offered by subjects with an in situ TIPSS to directly investigate the metabolic processing of folic acid and other folates by the intestinal tract. All subjects were in a program of follow-up monitoring and had stable liver cirrhosis. The physical location of the TIPSS (**Figure 1**) allows safe blood sampling from the hepatic portal vein, thereby providing a unique insight into the metabolic fate of folates immediately after passing through mucosal cells.

To be eligible for the study, participants had to have stable, synthetic liver function without recent evidence of decompensation (defined as liver-function inadequacy or active complications of portal hypertension), be abstinent from alcohol, be free from malignant disease, have normal gut permeability as evidenced by the recovery of urinary lactulose and mannitol after an oral test dose in the reference range, and have a patent TIPSS on their last surveillance. Participants who were receiving folic acid supplementation or taking vitamin B supplements were excluded. Six subjects who had undergone a TIPSS insertion at the Freeman Hospital, Newcastle on Tyne, United Kingdom, between 1992 and 2009 provided written, informed consent to take part in the study. All studies were conducted according to the guidelines laid down in the Declaration of Helsinki, and all procedures involving human subjects were approved by the Newcastle and North Tyneside 1 Research Ethics Committee (08/H0906/82). The small sample size was a consequence of the number of patients who were eligible in the hospital database (*n* = 26). Of these subjects, 17 patients did not return their expression of interest letter after one reminder. Of the other 9 subjects, one patients did not consent, one patient developed severe cervical arthritis and could not lie flat, and one patient had a neuropsychiatric illness during the screening phase.

The crossover study design allocated subjects with an in situ TIPSS to randomly receive either a physiologic 500-nmol (220 μg folic acid equivalent) dose of 13C5-folic acid or 13C5-6S-5-FormylTHF (Merck Eprova), with the label being carried by the 5 carbons of the glutamate moiety. Study days coincided with the participant's annual TIPSS surveillance checkup with crossover dosing that occurred at the next annual checkup. After an overnight fast, a routine TIPSS venogram was carried out by experienced radiologists to confirm the patency of the TIPSS. A catheter (65-cm 5Fr Beacon Tip Royal Flush Plus High Flow Catheter; Cook Medical Europe Ltd) was placed in the portal vein and flushed with 10 IU/mL heparin sodium solution (Hepsal Wockhardt UK Ltd). The position of the portal catheter was confirmed at the end of the procedure by using fluoroscopy. A peripheral venous cannula was placed in a median cubital fossa vein and flushed with a 0.9% sodium chloride solution.

Oral doses stored in a cold chain at −20°C were reconstituted in 30 mL sterile water (<0.25 EU/mL per Medicines and Healthcare Products Regulatory Agency guidelines), and timed (postdose) portal (15, 25, 35, 45, 55, 65, and 85 min) and systemic (30, 60, 90, 120, 150, 180, 210, and 240 min) venous blood samples were taken. The time frame of measurements was a result of limitations on the total volume of blood that could be drawn as stipulated by the Ethics Committee and also to keep it as reasonable as possible for the volunteers.

### Sample analysis

All blood samples were collected in 5-mL lithium heparin evacuated tubes (BD), which were held at 4°C before plasma preparation by using chilled centrifugation. Samples were stored in light-protected Eppendorf Safe-Lock tubes (Eppendorf UK Limited) at −80°C before analysis by using a high sensitivity liquid chromatography–tandem mass spectrometry assay on the basis of a method described previously (23). In brief, each sample (5 μL) was analyzed by using an Agilent 1200 binary HPLC (Agilent Technologies) coupled to an AB Sciex 4000 Qtrap triple-quadrupole mass spectrometer (AB Sciex UK Limited). HPLC was achieved by using a binary gradient of solvent A [MilliQ Water (VWR International Limited) plus 0.1% formic acid] and solvent B (HPLC-grade acetonitrile plus 0.1% formic acid) at a constant flow rate of 250 μL/min. Separation was made by using a Phenomenex Kinetex 2.6-μ C18 100 × 2.1-mm column (Phenomenex) maintained at 50°C. Injection was made at 2% B and held for 2.5 min, ramped to 10% B to 6 min, and ramped to 43% B by 15 min. A 98% B column wash was applied until 23 min, and the column was equilibrated to initial conditions for 10 min.

The mass spectrometer was operated in electrospray positive mode to monitor specific parent/fragment transitions for folate target compounds as folic acid (442/295), 13C-folic acid (447/295), 5MeTHF (460/313), 13C-5MeTHF (465/313), 5-FormylTHF (474/327), 13C-5FormylTHF (479/327), and methotrexate (455/308). Optimized ionization and collision energies were tuned and applied to each transition (not reported). Quantification was applied with Analyst 1.5 software (AB Sciex) to integrate detected peak areas relative to the methotrexate internal standard. Reproducibility (expressed as the CV) was as follows: folic acid = 8%, 13C-folic acid = 14%, 5MeTHF = 10%, 13C-5MeTHF = 7%, 5-FormylTHF = 13%, and 13C-5FormylTHF = 15%. Recovery was as follows: folic acid = 82%, 13C- folic acid = 94%, 5MeTHF = 77%, 13C-5MeTHF = 87%, 5-FormylTHF = 99%, and 13C-5FormylTHF = 85%.

### Statistical analysis

The percentage of folate in unmodified and modified forms in the portal vein for each of the different oral dosing formulations, at the initial 15-min postdosing time point was compared by using a paired Student's *t* test (Excel 2010; Microsoft Corp). The percentage of folate in modified and unmodified forms between different oral dosing formulations was compared by using an unpaired (2-sample) Student's *t* test. Although the sample size was small, diagnostic checks suggested that the data were appropriate for parametric statistical analysis methods. Data are presented as means ± SDs unless stated otherwise.

## RESULTS

Six subjects (4 men and 2 women) participated in the study between April 2009 and September 2011. Volunteer characteristics are given in **Table 1**. The age of participants was 55 ± 7 y, and BMI (in kg/m^2^) was 33 ± 10. The serum vitamin B-12 concentration was 555 ± 186 ng/L, and the red blood cell folate concentration was 698 ± 523 nmol/L. Measures of liver disease severity were monitored (eg, by using the Childs-Pugh Score and Grade and MELD Score), and results indicated that study participants had little or no ongoing liver injuries (data not shown). Of 6 subjects that entered the study, all subjects completed the folic acid arm, but subjects 4 and 5 did not ingest the labeled 5-FormylTHF dose because their annual TIPSS checkups fell outside the time frame of the study. Therefore, results are reported as *n* = 6 for folic acid and *n* = 4 for 5-FormylTHF unless stated otherwise.

**TABLE 1:** Baseline characteristics of study participants at study enrollment*^1^*

Subject identifier	Sex	Age	Etiology of liver disease	BMI	Plasma B-12 concentration	Red blood cell folate concentration
		*y*		*kg/m^2^*	*ng/L*	*nmol/L*
1	M	57	Nonalcoholic steatohepatitis	33	806	1679
2	M	56	Alcohol	42	576	888
3	M	61	Alcohol	27	336	326
4	F	59	Alcohol	26	569	342
5	M	44	Alcohol	47	349	419
6	F	53	Budd-Chiari	23	692	535

1 All participants were clinically stable, and there was no significant change in any variable, and no deterioration in any variable was seen between annual assessments.

**FIGURE 2.**
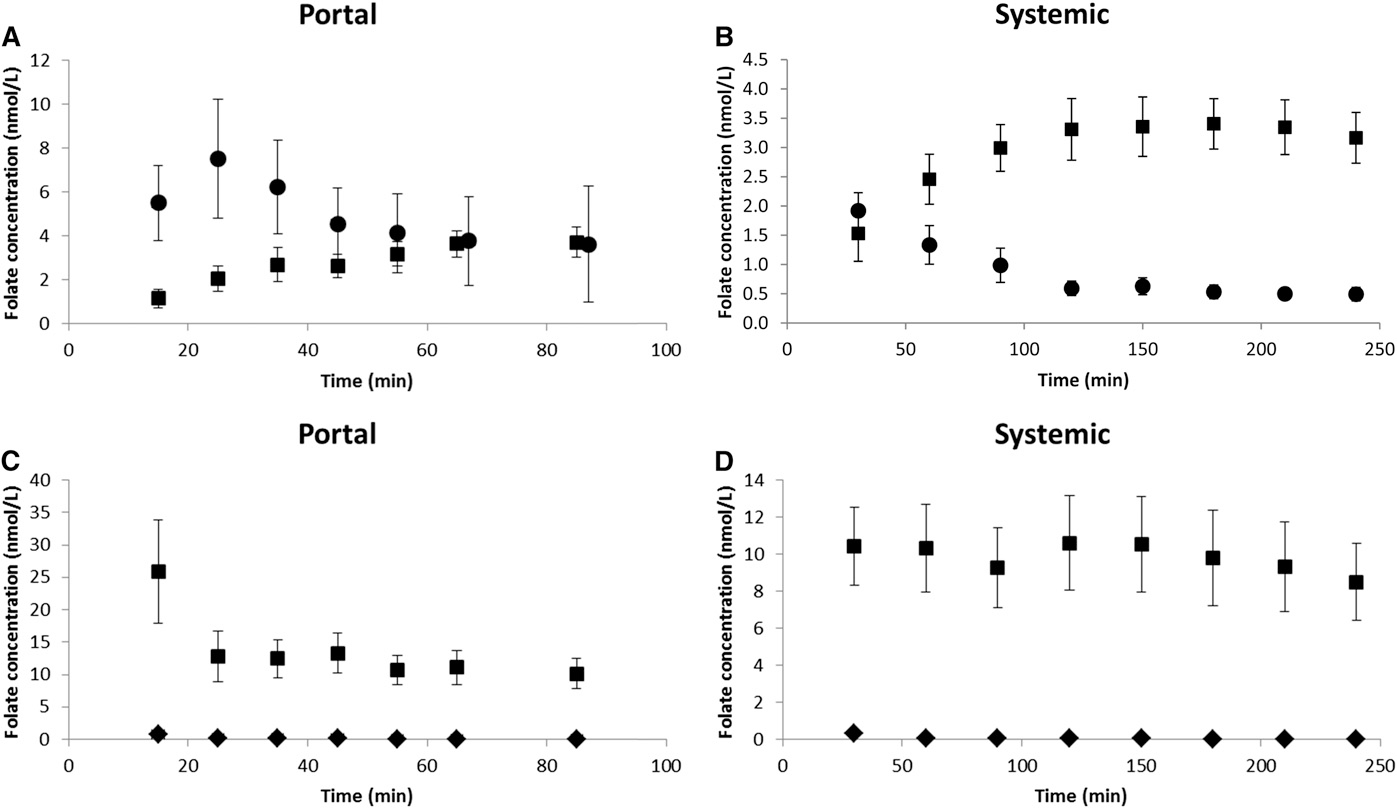
A: Mean (±SEM) concentrations of labeled folic acid (circles) and labeled 5-MTHF (squares) in the hepatic portal venous circulation after the ingestion of a labeled folic acid dose (*n* = 6). At time = 15 min, there was a significantly (*P* < 0.01) higher percentage of labeled folic acid than labeled 5-MTHF. B: Mean (±SEM) concentrations of labeled folic acid (circles) and labeled 5-MTHF (squares) in systemic plasma after the ingestion of a labeled folic acid dose (*n* = 6). C: Mean (±SEM) concentrations of labeled 5-FormylTHF (diamonds) and labeled 5-MTHF (squares) in the hepatic portal venous circulation after the ingestion of a labeled formyl dose (*n* = 4). At time = 15 min, there was a significantly (*P* < 0.001) higher percentage of labeled 5-MTHF than of labeled 5-FormylTHF. D: Mean (±SEM) concentrations of labeled 5-FormylTHF (diamonds) and labeled 5-MTHF (squares) in systemic plasma after the ingestion of a labeled formyl dose (*n* = 4). Note differences in study durations for portal (A and C) and systemic (B and D) elements of the study. Also note differences in *y*-axis scales between panels. 5-FormylTHF, 5-formyltetrahydrofolic acid; 5-MTHF, 5-methyltetrahydrofolic acid.

The comparison of portal venous plasma concentrations of labeled folic acid and labeled 5-MTHF (the product of the physiologic reduction and methylation of dietary folates) after ingestion of the folic acid dose showed that unmetabolized labeled folic acid concentration rose significantly and more rapidly than did labeled 5-MTHF concentrations (**Figure 2A**). At the initial 15-min sampling time point, a consistent pattern was seen across 5 participants (one volunteer's 15-min sample did not contain any detectable labeled folate) with 80 ± 12% of labeled folate in the hepatic portal vein after a dose of folic acid being unmodified folic acid. This result was significantly higher (*P* < 0.01) than the 20 ± 12% for labeled 5-MTHF. The labeled folic acid concentration peaked at ∼25 min (postdose), and steadily declined to be approximately equal in concentration to the labeled 5-MTHF at 85 min. In contrast, the labeled 5-MTHF concentration rose slowly from 1.2 ± 4.2 nmol/L at 15 min to a maximum of 3.7 ± 6.5 nmol/L at 85 min. The appearance of labeled folic acid was also observed in the systemic (peripheral) circulation at each time point (Figure 2B).

In contrast, at the initial 15-min time point, the unmodified form of labeled 5-FormylTHF was seen at extremely low concentrations in the portal circulation when subjects were given the 5-FormylTHF dose (Figure 2C). A mean (±SD) 4 ± 18% of labeled folate in the hepatic portal vein was unmodified 5-FormylTHF compared with 96 ± 18% for labeled 5-MTHF (*P* < 0.001). The labeled 5-FormylTHF concentration was very low and had a maximum value at 15 min (0.8 ± 0.4 nmol/L) before steadily declining to ∼0.1 ± 0.1 nmol/L at 85 min. In contrast, the labeled 5-MTHF concentration was high and peaked at 15 min with a mean (±SD) of 25.9 ± 15.9 nmol/L and declined steadily to 10.1 ± 4.7 nmol/L at 85 min. Very little of the unmodified form of 5-FormylTHF was observed in the systemic plasma at any time point (Figure 2D).

When we compared between oral doses, the percentage of total labeled folate that appeared as unmodified folic acid in the hepatic portal vein was significantly greater (*P* < 0.0001) than the percentage of total labeled folate that appeared as unmodified 5-FormylTHF at each time point. In addition, at the 15-min time point, the mean concentration of labeled 5-MTHF (1.2 ± 1.0 nmol/L) derived from folic acid was significantly lower (*P* < 0.01) than the mean concentration of labeled 5-MTHF (25.9 ± 15.9 nmol/L) derived from 5-FormylTHF.

The AUC of the data presented in Figure 2, A and C, can be used as a surrogate measure for the quantity of labeled folate entering the portal blood systems from folic acid and 5-FormylTHF doses, respectively. The AUC was estimated by using the trapezoidal method (or rule). In brief, the area bounded by the* y* axis, *x* axis, and curve was divided into rectangles, and the areas of those rectangles (base × height) were calculated, and each rectangle area was summed to arrive at the estimate for the AUC. From Figure 2A, it can be calculated that, for the oral dose of folic acid, the AUC_Folicdose_ of total labeled folate (folic acid + 5-MTHF) was 597 nmol · min^−1^ · L^−1^. From Figure 2C, it can be calculated that, for the oral dose of 5-FormylTHF, the AUC_Formyldose_ of total labeled folate (5-FormylTHF + 5-MTHF) was 1102 nmol · min^−1^ · L^−1^. The ratio of the 2 (AUC_Formyldose_:AUC_Folicdose_) was 1.8. The results indicated that almost twice as much folate from the labeled 5-FormylTHF dose crossed into the hepatic portal vein from mucosal cells in the first 85 min (postdose) compared with the amount of folate derived from the labeled folic acid dose.

In addition, the maximum concentration over the timeframe of the experiment of labeled 5-MTHF in the hepatic portal vein was seen to occur 85 min after the ingestion of the folic acid dose (Figure 2A) compared with that just 15 min after the dose of 5-FormylTHF (Figure 2C). The magnitude of these maximum concentrations of labeled 5-MTHF were significantly different [3.7 ± 1.7 nmol/L (after the folic acid dose) compared with 25.9 ± 15.9 nmol/L (after the 5-FormylTHF dose); *P* < 0.001].

## DISCUSSION

In this study, it has been shown that the majority of a physiologic oral dose of folic acid passes into the portal venous circulation in an unmodified form. In contrast, the oral dose of the dietary folate 5-FormylTHF was nearly all converted and appeared in the portal venous circulation almost entirely as 5-MTHF, which is an observation that confirmed normal gut wall function in terms of both gut permeability and the folate methylation capacity.

These findings fundamentally challenge the model of folic acid biotransformation when applied specifically to humans. The reason for the very limited reduction and subsequent methylation of folic acid to 5-MTHF in mucosal cells was probably the inadequate activity of dihydrofolate reductase (DHFR) in the enterocyte, which made a reduction of folic acid the rate-limiting step. A recent study showed that the human liver has a low and highly variable DHFR activity (24). If this finding was replicated in human mucosal cells, it would explain the current study observation of unmodified folic acid in the portal vein.

The most obvious reason for the discrepancy between findings in this study and those in previous studies that showed the gut reduction and methylation of folic acid was the use of rodent models (most typically rat) and human cell lines. Both of these approaches have inherent weaknesses. Rats have significantly higher concentrations of DHFR than do humans (24), which make rats a poor model for human folic acid metabolism. Human cell lines, grown in vitro, also exhibit elevated concentrations of DHFR activity compared with that of human tumors or cells obtained in situ, potentially as a consequence of the traditional use of high concentrations of folic acid in tissue culture medium (25).

Findings in this study confirmed and extended those of earlier human studies published in the 1960s and 1970s (26, 27). These articles were criticized for administering large, nonphysiologic doses of folic acid and were largely disregarded. However, it was a pertinent finding that no dose-derived 5-MTHF was initially detected in the hepatic portal vein, albeit one overlooked by later researchers.

Another surprising finding from the current study was the apparent lower absorption of folic acid compared with 5-FormylTHF as evidenced from an examination of Figure 2, A and C, and the computation of respective AUCs of the total labeled folate that appeared in the portal vein during the first 85 min postdose. Nearly 2 times more labeled folate appeared to cross the gut wall from the ingestion of the 5-FormylTHF dose compared with that from the folic acid dose. At face value, this finding appears to contradict previous studies in ileostomy subjects that showed that single 453-nmol doses of 13C5-folic acid (close to the 500-nmol dose in the current study) were extensively absorbed (∼90%) by the gut mucosa (28). Also, daily supplementation with either folic acid or 5-MTHF has been equally effective in raising red blood cell folate concentrations to the same extent (29).

**FIGURE 3.**
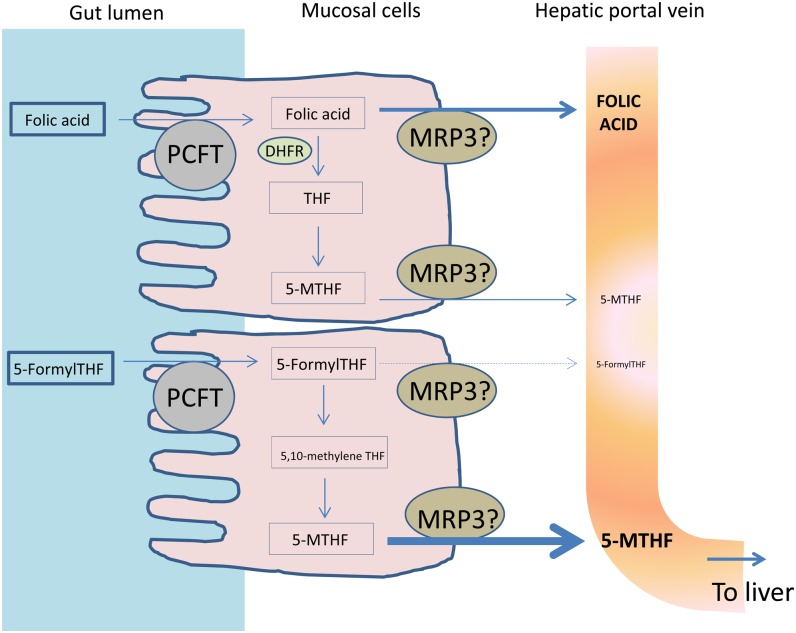
Proposed folate absorption from the gut lumen, metabolism in mucosal cells, and transport out into the hepatic portal vein. DHFR, dihydrofolate reductase; MRP3, multidrug resistance protein 3; PCFT, proton-coupled folate transporter; THF, tetrahydrofolate; 5-FormylTHF, 5-formyltetrahydrofolic acid; 5-MTHF, 5-methyltetrahydrofolic acid.

A possible explanation for the apparent discrepancy between the low absorption of folic acid as calculated from the AUC of total labeled folate that initially appeared in the portal vein in the current study and that calculated from ileostomy subjects can be ascertained by examining the proposed mechanism of folate transport into and out of the enterocytes (shown schematically in **Figure 3**). First, both reduced tetrahydrofolates (eg, 5-FormylTHF) and folic acid are taken up by mucosal cells with a similar affinity by the proton-coupled folate transporter (PCFT) (16). Once inside the mucosa, the cell machinery attempts to metabolize the newly absorbed folate to 5-MTHF. In the case of folic acid, this metabolization appears to be extremely limited. Finally, the unmetabolized and metabolized folate will be transported out of the cell into the hepatic portal vein. The mechanism of folate transport out of the mucosal cells is not clear, but multidrug resistance proteins (MRPs) are expressed at the basolateral membrane, and it is thought that they have a key role to play, especially that of MRP3 (16). However, MRP3 transports folic acid with a much-lower efficiency than the reduced tetrahydrofolate form (30). This lack of efficiency, rather than the different extent of absorption, might explain the observation that approximately twice as much labeled folate was shown in the portal blood after the 5-FormylTHF dose than the folic acid dose during the first 85 min postdose.

In this study, all subjects had inactive liver disease and normal liver synthetic function and all were abstinent from alcohol. The major confounding factor, ie, that of increased gut permeability, was eliminated both by prescreening for gut permeability (data not shown) and determining, as part of the study, the complete conversion of the dose of natural folate (5-FormylTHF). The essential absence of 5-FormylTHF detected in the hepatic portal vein following a dose of 5-FormylTHF implies that the passage of all labeled folate was through the enterocytes and not between enterocytes. Therefore, the pattern of data that is shown in Figure 2, A and C, generated from the analysis of the hepatic portal vein blood samples should reflect that shown in the general population.

More caution needs to be applied to systemic circulation data (Figure 2, B and D) because of the capacity for periportal shunting effects in TIPSS patients. The subsequent appearance of unmodified folic acid in the peripheral circulation reflected the medical design of the TIPSS; significant quantities of blood from the hepatic portal vein bypassed the liver via a shunt to the hepatic vein (and then onto the inferior vena cava as shown in Figure 1).

When single physiologic 634-nmol (280-μg) doses of stable-isotope–labeled folic acid have been given to subjects without a TIPSS, who were neither exposed to mandatory fortification nor self-supplementation, no unmetabolized labeled folic acid was seen in the systemic circulation, only labeled 5-MTHF (22). However, note that the use of a combined HPLC and microbiological assay, subnanomolar concentrations were shown in 1 of 4 subjects given 5 consecutive doses of 200 μg (453 nmol) folic acid at 90-min intervals (31).

If folic acid (when given as a single physiologic dose <634 nmol) is mainly transferred to the hepatic portal vein unmetabolized, it may be concluded that, under normal experimental conditions, folic acid that enters the hepatic portal vein must be almost completely removed by the liver during its first pass and, because of its low DHFR activity, only slowly biotransformed to 5-MTHF before either entering the systemic circulation in a visibly attenuated response or being excreted in the bile. It is also possible that some folic acid may be converted to polyglutamate forms and stored in the liver. Whatever the initial fate of the folic acid, the human liver has been shown to have not only a low but highly variable DHFR activity (24). Therefore, chronic exposure to folic acid in physiologic doses (as would be the case with mandatory fortification) may induce saturation and explain the observed systemic circulation of unmetabolized folic acid (8).

We acknowledge that the study was limited in terms of the number of subjects studied. However, this limitation reflects the unique and rare nature of the population in whom the study could be performed (ie, hepatologically stable patients with a TIPSS in situ). Despite the limitation of the study population size, we believe that the study of this unique group of people has allowed important insights into the physiology of folate absorption in humans that could not have been generated in any other experimental system.

In conclusion, a low enzyme activity of DHFR may compromise both mucosal and liver biotransformation of folic acid in humans. For dietary supplements, it is suggested that folic acid could be replaced with 6S-5-MTHF (the normal systemically circulating folate form), the multiple advantages of which have been noted previously (32). The Food and Drug Administration in the United States and the European Food Standards Agency have already approved products containing a 5-MTHF calcium salt (Metafolin; Merck & Cie) and a 5-MTHF glucosamine salt (Quatrefolic; Gnosis).

It is also suggested that effort is made to microencapsulate 5-MTHF so that losses from manufacture, the use in voluntary fortified foods (eg, breakfast cereals), and the subsequent processing (eg, heat) of products are minimized (33). If this effort can be accomplished successfully, consideration could be given to replacing folic acid with 5-MTHF in countries that have mandatory programs of flour fortification.

## Footnotes

4 Abbreviations used: DHFR, dihydrofolate reductase; TIPSS, transjugular intrahepatic porto systemic shunt; 5-FormylTHF, 5-formyltetrahydrofolic acid; 5-MTHF, 5-methyltetrahydrofolic acid.
